# Fascia Mobility, Proprioception, and Myofascial Pain

**DOI:** 10.3390/life11070668

**Published:** 2021-07-08

**Authors:** Helene M. Langevin

**Affiliations:** National Center for Complementary and Integrative Health, National Institutes of Health, 31 Center Drive, Suite 2B11, Bethesda, MD 20892, USA; helene.langevin@nih.gov; Tel.: +1-301-435-6826

**Keywords:** fascia, interoception, proprioception, myofascial pain, connective tissue, fascia mobility

## Abstract

The network of fasciae is an important part of the musculoskeletal system that is often overlooked. Fascia mobility, especially along shear planes separating muscles, is critical for musculoskeletal function and may play an important, but little studied, role in proprioception. Fasciae, especially the deep epimysium and aponeuroses, have recently been recognized as highly innervated with small diameter fibers that can transmit nociceptive signals, especially in the presence of inflammation. Patients with connective tissue hyper- and hypo-mobility disorders suffer in large number from musculoskeletal pain, and many have abnormal proprioception. The relationships among fascia mobility, proprioception, and myofascial pain are largely unstudied, but a better understanding of these areas could result in improved care for many patients with musculoskeletal pain.

## 1. Introduction

Anyone who has participated in a yoga class knows that some people are more “flexible” than others. We usually think of musculoskeletal flexibility as “loose” or “tight” joints—ligaments, joint capsules—and muscles. But another important part of the musculoskeletal system that tends to be overlooked is the network of fasciae. These are sheets of connective tissue that form interconnecting planes spanning the entire body, surrounding and separating muscles, and creating biomechanical interfaces between them.

Fasciae are composed of irregularly arranged but tightly woven connective tissue that can bear high tensile loads [1,2]. Fascial planes are separated by planes of “loose” connective tissue that allows the fasciae to glide past one another [1,3]. The motion between the fascia layers is a significant component of musculoskeletal mobility. When two adjacent layers become adherent, either due to scarring after an injury, or due to posture habits, some of the interfacial mobility is lost. The consequences of reduced fascia mobility on the function of muscles and joints are potentially profound, but mostly unknown.

Furthermore, the sensory information derived from fasciae and its contribution to proprioception (internal “body-sense”) and musculoskeletal pain is basically unstudied. This is because, historically, more attention has been given to specialized musculoskeletal tissues (i.e., bones, cartilage, intervertebral discs, muscle) than to “non-specialized” connective tissue, including fasciae, but this is beginning to change.

Although the clinical syndrome of “myofascial pain” remains poorly characterized, it is estimated to be present in approximately 30% of patients with chronic musculoskeletal pain [4,5] in the back, neck, shoulder, hip, and pelvis as well as temporomandibular pain, and headache. Over the past two years, three National Institutes of Health (NIH) workshops have addressed these related topics to stimulate research: “Quantitative Evaluation of Myofascial Tissues” [6], “Neurocircuitry of Force-Based Manipulation” [7], and “The Science of Interoception and Its Roles in Nervous System Disorders” [8].

This article summarizes what we currently know about fascia mobility and proprioception and their potential relationship to myofascial pain, while identifying knowledge gaps that need to be filled.

## 2. Fasciae, Proprioception, and Interoception

Fasciae are part of a network that is given different names as one moves from the cellular level, to tissues, organs, and the whole body: extracellular matrix, interstitium, connective tissue, and fasciae. All connective tissue consists of both cellular and extracellular components, and it is the varying molecular and architectural characteristics of these extracellular constituents that determine their mechanical properties. Some types of connective tissue (e.g., areolar subcutaneous tissue) are loosely organized and compliant, while others (e.g., deep epimysium) are tightly woven and stiffer [2].

Until recently, there was very little knowledge on the innervation of connective tissue. It is now clear that at least the deep muscular fasciae and aponeuroses are known to be richly innervated with small-diameter afferent fibers that can transmit nociceptive signals [9,10,11]. What remains unknown is the degree to which connective tissue contributes to nonpainful sensations such as those experienced during deep pressure and stretching, and the degree to which sensations arising from it contribute to proprioception and interoception.

Interoception is the processes by which the body senses, interprets, integrates, and regulates signals from within itself, and includes sensations arising from connective tissue deep to the skin. This definition includes proprioception in its broadest sense, meaning the sensory perception and awareness of the position and movement of the body [12]. Clinically, however, proprioception is defined more narrowly and evaluated as the ability to sense whether a joint is moving in one direction versus another. We know that this ability is dependent on the functioning of fast-adapting specialized mechanoreceptors in joint capsules and tendons [13]; however, there may be other important aspects of proprioception beyond joint position sense.

One way to demonstrate this is to grab your forearm and squeeze it (deep pressure) and then twist until you feel some resistance (stretching) and notice the experienced sensations from deep tissues—interstitial and fasciae. These sensations do not arise from joint-associated structures, yet they clearly play a role in everyday sensations that are experienced when moving and changing posture. It has been proposed that mechanical input arising from the deformation of deep tissues, not just the skin, is an important component of the perceptual haptic systems by which one perceives the external surfaces adjacent to it [14]. Sensations arising from deep tissues are particularly relevant to activities that involve movement beyond one’s habitual range of movement, such as stretching and yoga. Importantly, such sensations of deep pressure and stretching persist when the stimulus is sustained, and therefore must involve yet-to-be-discovered slow-adapting mechanosensory neurons.

At the cellular level, several types of ion channels are activated by mechanical stimuli, and among these, Piezo channels have been shown to be responsible for the sensation of light touch (skin) and joint position sense [15]. In humans, there is also recent evidence that A-β sensory fibers, but not Piezo2 channels, are required for pleasant, nonpainful deep pressure sensation, as experienced during a massage [16]. However, it remains unknown what type of sensory fibers or mechanoreceptors are involved in deep-tissue stretching sensations.

Although we do not have a detailed map of the somatosensory areas of the brain that process sensations from fasciae, they play an important role in the overall sense of “inner body awareness” that involves brain areas linked to emotions, such as the insula and cingulate cortices [17]. The “de qi” deep tissue sensation evoked by stretching of connective tissue during acupuncture needling is another example of sensation that falls under the broad definition of interoception and has been shown to cause both activation of somatosensory areas and de-activation of limbic and paralimbic brain structures [18,19].

## 3. What Types of Stresses and Strains May Play a Role in Proprioceptive Signals from Fascia?

**Figure 1:**
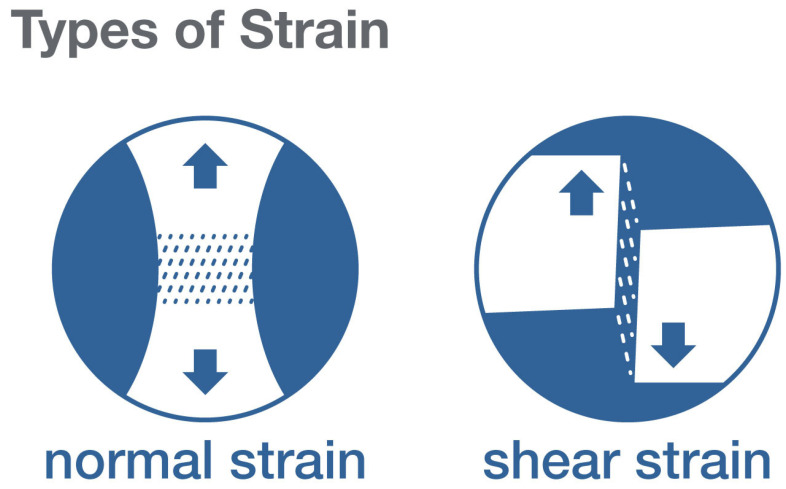
Types of strain resulting from normal and shear forces.

In biological tissues, mechanical forces influence a wide range of cellular and molecular processes, for example: “normal” forces aligned to each other and unaligned shearing forces that push parts of a body in opposite directions (Figure 1). Whether a cell or channel responds to an externally applied mechanical force or to local deformation, or strain, created within the tissue by the force, is an important question because the same amount of force will produce more or less deformation depending on the viscoelastic properties of the tissue. This becomes particularly significant for connective tissue, the structure of which varies widely from loosely organized to densely packed and can be influenced by pathological processes such as chronic inflammation and fibrosis [20].

**Figure 2:**
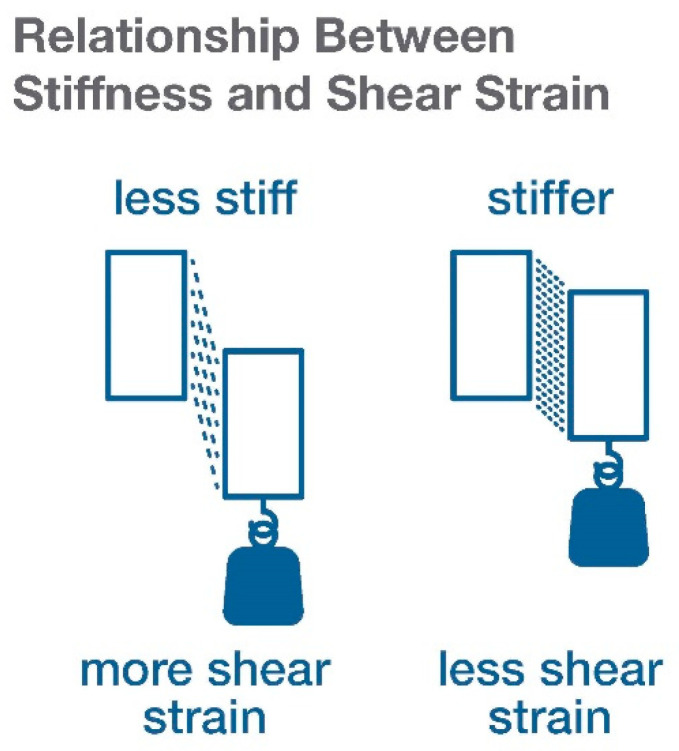
Relationship between stiffness and shear strain.

Biological tissues are viscoelastic, which means that their mechanical behavior can be described as that of both a solid (elastic) and a liquid (viscous) [21,22,23]. A fundamental concept of the elastic component is the relationship among stress (applied force), strain (deformation resulting from an applied force), and stiffness [24]. An easy way to visualize this relationship is to think about hanging the same weight from two rubber bands of different stiffnesses: the stiffer rubber band will stretch less. This also applies to the material properties of biological tissues: the same amount of force will produce more or less strain depending on its stiffness properties: the stiffer tissue will deform less, while softer tissue will deform more. For shear forces, the stiffness of the material forming the interface between two bodies will determine how much strain will develop within the interface as the two bodies move past one another (Figure 2).

Viscosity is also important for determining the effect of normal and shear forces and is related to the “liquid-like,” rate-dependent component of strain; that is, connective tissue will deform less when a force is applied quickly than when applied slowly, which allows the tissue structure to reorganize in a liquid-like manner as the force is applied [21]. In biological tissues, areolar connective tissue forms the interface between layers of fascia. A given amount of shear force will result in more or less strain depending on the stiffness and viscosity of the areolar layer, which is itself determined by its collagen density and cross-linking, glycosaminoglycan composition, and water content. Reversible “densification” of areolar connective tissue may occur with changes in hyaluronic acid concentration, pH, and temperature [25].

Mechanically activated ion channels (e.g., Piezo, transient receptor potential channels) are sometimes referred to as “force sensors” but may respond to the deformation (strain) of the cell membrane rather than the force [26]. If this is the case, then the response of the channel to an applied force would be expected to vary with alterations to the stiffness of its environment. This could include changes in the extracellular matrix, intracellular cytoskeleton, or membrane itself. This effect can be evaluated experimentally in vitro by using drugs to induce stiffening of the extracellular matrix (e.g., collagen crosslinking), cytoskeleton (e.g., colchicine), or membrane lipids (GsMTx-4). Indeed, a growing literature on mechanotransduction, mostly in the cancer field, suggests that normal or shear strain may be a major determinant of cellular responses [27].

## 4. Tissue Stress, Strain, and Stiffness Measurement In Vivo

The above considerations underscore the importance of characterizing tissue stress, strain, and stiffness when performing studies in vitro or in vivo. This is especially critical when studying pathological conditions that may be associated with changes in connective tissue composition and architecture either resulting from a clinical condition or experimental model like chronic inflammation or fibrosis, or treatment such as an anti-inflammatory drug or nonpharmacologic therapy. At the microscopic level, atomic force microscopy and magnetic twisting microscopy have been used to estimate local strain and stiffness within cells [28].

Measuring tissue stress and strain can be particularly challenging in vivo. Although there has been recent progress in developing methods to measure applied forces, it remains difficult to measure stress in tissue directly because stresses in various parts of non-homogeneous tissues can vary locally when external force is applied. On the other hand, there are methods to measure how strain is distributed through tissues in response to an applied force. Elastography is the quantitative imaging of strain distribution in soft tissue and was initially developed using the transducer of a B-scan ultrasound to compress the tissues externally [29,30]. Ultrasound elastography was developed to measure the stiffness of breast and prostate cancer lesions but has since been used in a variety of other clinical applications [31,32]. Importantly, ultrasound elastography based on tissue compression does not directly measure elasticity or stiffness; rather, it calculates local elastic modulus profiles based on assuming a uniform stress distribution through the tissue. This has obvious limitations for musculoskeletal tissues with complex structures. Related methods such as shear-wave ultrasound elastography and magnetic resonance elastography (MRE) have used other types of mechanical input to estimate tissue strain and stiffness at the macroscopic level [33].

Measuring tissue strain in vivo is particularly important, and challenging, when strain occurs in shear, which is the case for layered myofascial tissues that normally glide past one another during normal body movements [1]. The layers of loose and dense connective tissue that surround and separate muscles create multiple interfaces where shear strain occurs as the tissues are passively moved due to an external force, or actively moved by muscle contractions. Pathological processes such as inflammation, fibrosis and scarring can cause layers to adhere to one another and reduce shear strain. An example of this was demonstrated in human subjects with chronic low-back pain who were shown to have reduced shear strain within layers of the thoracolumbar fascia compared with control subjects without low-back pain [3]. In pigs, similar fasciae adhesions and shear strain reduction were produced experimentally using simple movement restriction [34].

Studies using ultrasound or other imaging techniques such as magnetic resonance elastography (MRE) could investigate this further. MRE can be used to examine the mechanical properties of increased stiffness, altered viscosity and reduced strain, as well as the structural features of scarring, fibrosis, and disorganized architecture, and also could potentially quantify the degree of adhesion at functional myofascial interfaces [35]. Recent studies using T1ρ MR imaging suggested that high levels of unbound water, indicating hyaluronan aggregation, may also contribute to increased stiffness and reduced mobility of fasciae [36]. Whatever the scale (micro to macro) of the experiment, quantifying fascia mobility is important for understanding proprioceptive and other signals generated by mechanical stimuli on tissues and cells, especially when pathological conditions affect the stiffness of the tissue.

## 5. Fascia Mobility and Myofascial Pain

**Figure 3:**
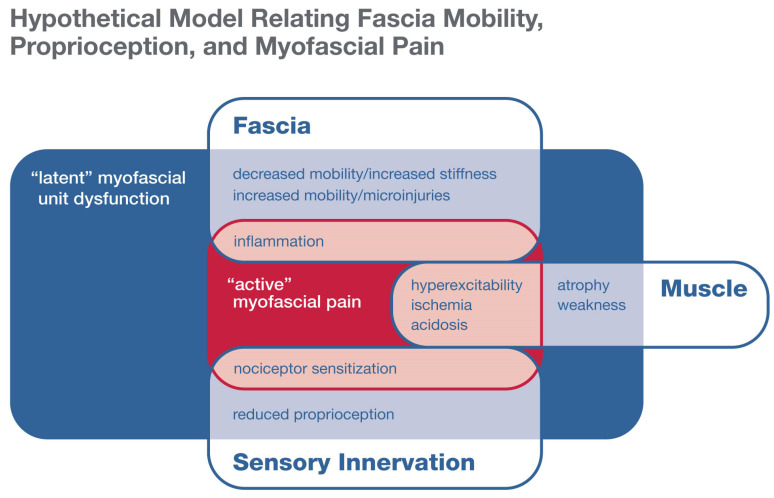
Hypothetical model relating fascia mobility, proprioception, and myofascial pain.

In addition to being important in proprioception, fascia mobility may be a key component of myofascial pain, a poorly understood but extremely common condition [4,5]. Fasciae, especially epimysial and aponeurotic, have recently been recognized as highly innervated with small-diameter fibers that can transmit nociceptive signals, especially in the presence of inflammation [9,10,11,37]. Likewise, myofascial tissues are now receiving increasing attention as possible “pain generators”. Since the 1960s, the clinical literature has included descriptions of myofascial pain syndrome, which is currently diagnosed based on signs and symptoms, including the palpation of tender, indurated nodules termed myofascial “trigger points” [38,39]. Myofascial pain syndrome is distinguished from fibromyalgia by its more focal and episodic nature and includes both “active” and “latent” phases [40] (Figure 3). The active phase is characterized by spontaneous pain that limits the range of motion, with localized, tender indurated foci within the myofascial tissues. Palpation of these nodules reproduces the patient’s pattern of local or radiating pain. During the latent phase, palpable focal nodules are present and may be tender but without spontaneous pain. The latent phase also includes myofascial dysfunction, including soft tissue stiffness and reduced range of motion (not associated with pain) in the affected area. The increased soft tissue stiffness and movement restriction could reflect focal areas of persistent muscle contraction or connective tissue adhesions that locally restrict shear plane movement or shear strain [41].

Possible pathophysiological mechanisms involving the myofascial unit—muscles and associated connective tissue, nerves, blood vessels, and lymphatics—that may contribute to myofascial pain include neurogenic and chronic inflammation, peripheral sensitization, muscle hyperexcitability, ischemia, and acidosis [40] (Figure 3). Several of these mechanisms may involve reduced or increased fascia mobility. Chronic inflammation with macrophage infiltration and fibrosis of deep fasciae and perineural tissues has been well documented in an animal model of repetitive motion injury [42]. Because fasciae are organized in layers, an important effect of fibrosis is adhesion between the layers with reduced shear strain.

One of the factors that has limited the understanding of myofascial pain has been the lack of imaging and other objective methods to quantify pathological abnormalities of the myofascial unit. For many years, structural imaging (X-rays, computerized tomography, magnetic resonance imaging) of “hard” tissues (bones, cartilage, intervertebral discs) were the only tools to evaluate patients with musculoskeletal pain. Over time, it became apparent that these biomarkers lacked sensitivity and specificity. For example, in low-back pain, many asymptomatic patients have abnormal-looking discs, and a large number of patients with severe, disabling back pain have no demonstrable structural abnormalities in the spine [43,44]. Meanwhile, imaging and other objective measurements of soft tissues (including muscles, and especially connective tissue) lag far behind, but are gaining recognition as possible musculoskeletal pain biomarker candidates [45,46].

A recent NIH workshop outlined state-of-the-art imaging technologies with some preliminary data on myofascial tissues, other types of technology (imaging, electrophysiology, metabolic measurements) used in musculoskeletal tissues but not yet for myofascial pain, as well as other technologies used in other tissues (brain, liver) that could be applied to musculoskeletal tissues [6]. Thus, there is an opportunity to gain ground through a multidisciplinary approach to harness these technologies for the development of quantitative diagnostic and predictive biomarkers of myofascial pain. The workshop also explored the potentials of incorporating tissue engineering, artificial intelligence, and computational modeling into the nascent research on myofascial pain.

## 6. Does Generalized Hypo- or Hypermobility Predispose One to, or Protect from, Myofascial Pain?

A window of opportunity for understanding the relationship between connective tissue mobility and myofascial pain comes from a variety of inherited and acquired connective tissue disorders with reduced or increased tissue mobility in a wide spectrum of phenotypic severity as well as a high prevalence of musculoskeletal pain.

Connective tissue disorders with reduced fascia mobility include inherited conditions that cause increased connective tissue thickness, such as acromelic dysplasias (caused by mutations in genes that encode secreted extracellular matrix proteins and proteins involved in TGF-β signaling), progerias and other premature aging syndromes like Werner’s, which features increased collagen I and III production [47]. Non-inherited disorders featuring reduced connective tissue mobility include scleroderma and scleroderma-like syndromes such as chronic graft-versus-host-disease (GVHD), hypothyroidism, renal failure, and diabetes [48]. Among these conditions, scleroderma and GVHD have been the most studied for musculoskeletal pain. In a recent cross-sectional study of 537 patients with scleroderma in five European countries, 80–92% reported joint pain, and nearly as many (70–86%) reported “muscle” pain [49]. Although it is not clear what role, if any, fascia adhesions may play in these symptoms, a murine model of GVHD demonstrated reduced shear plane motion between fascia layers, similar phenotypically to the reduced fascia mobility observed in patients with chronic low-back pain [50].

Conditions with increased fascia mobility include Ehlers-Danlos Syndromes (EDS) and Hypermobility Spectrum Disorders (HSD), a group of conditions involving connective tissue that can be inherited and are generally characterized by joint hypermobility, with or without skin hyperextensibility, and tissue fragility. Patients with EDS have high rates of musculoskeletal pain affecting the back and large joints (shoulder, knee, and hip), suggesting that the larger, higher activity-bearing joints with greater ranges of motion may be more susceptible to injury and pain, with a lower prevalence of pain in smaller, less mobile joints [51,52]. Although most of the known musculoskeletal manifestations of these syndromes relate to joints, the involvement of other connective tissues, including fasciae, may be relevant to the frequent occurrence of chronic musculoskeletal pain in these patients. Although myofascial pain syndrome is thought to be very common in patients with EDS/HSD, its exact prevalence is unknown due to a lack of objective methods to evaluate myofascial tissues. Lack of objective measurements also impairs research into the efficacy of treatments.

A number of well-established, as well as newly discovered, biomechanical features of connective tissue are relevant to the question of whether fascia hyper-or hypomobility influences myofascial pain. When a muscle contracts and begins to transmit a load to a tendon, an initial deformation (strain) will occur in the tendon before it fully bears the load (corresponding to the “toe region” of the stress-strain curve), and the amount of strain will depend on the stiffness of the tendon [24]. When a tendon is less stiff, the muscle it’s attached to needs to work harder and shorten more to make the connective tissue taut and become mechanically coupled to the joint. Furthermore, when working at higher strains, connective tissues are closer to the failing part of the stress-strain curve and become more prone to injury. In addition, it is now understood that a substantial percentage of the force exerted by a muscle is not transmitted to the tendon, but rather to adjacent muscles through the layers of fascia [53,54]. This implies that before a force can be transmitted, fasciae need to become mechanically coupled in shear. If interfascial connective tissue is loose and hypermobile, force may not be transmitted to the fascia if the stress-strain curve is still in the toe region. If interfascial connective tissue is stiff and hypomobile, the adjacent tissues will become mechanically coupled sooner, and the muscles will lose independent range of movement. Another important consequence is that if shear strain between layers of fascia is reduced, the layers can adhere and lose mobility altogether. Therefore, myofascial force transmission may vary depending on whether a person’s connective tissue is hypermobile or hypomobile.

Patients also may have generalized hypermobility but superimposed focal hypomobility in areas of scarring or adhesions due to macro- or micro-injuries. Thus, the pathogenic mechanisms of myofascial pain may be different in hypermobile versus hypomobile tissues. Clearly, more studies are needed to examine the role played by fascia mobility (reduced or increased) in musculoskeletal pain, and increased knowledge in this area could not only improve the lives of patients with inherited and acquired connective tissue disorders but also illuminate the role of fasciae and fascia mobility in musculoskeletal pain.

## 7. What Role Could Proprioception Play in Connective Tissue Hyper- or Hypomobility Syndromes?

At present, it is unclear what role abnormal proprioception may play in the pathogenesis of hyper- or hypomobility syndromes. It is well documented that patients with EDS/HSD have proprioception abnormalities [55]. These could be secondary to increased mobility, or pain could be interfering with proprioceptive acuity. It is also possible that these abnormalities are part of the mechanism of some hypermobility syndromes. Reduction in proprioception may, therefore, be attributed to impaired feedback mechanisms, pain, or a combination of both.

Some human genetic disorders involving mechanosensory channels may shed light on this question. Piezo channels are mechanosensory ion channels that are important for joint proprioception [13] although their presence/role in fascia is currently unknown. Piezo2 deficiency syndrome (a form of arthrogryposis) is characterized by both joint hypermobility and contractures as well as muscle weakness. Patients with Piezo gain- or loss-of-function mutations have not been systematically investigated for connective tissue biomechanical properties although there are reports of joint laxity in individual patients and families [56,57]. The coexistence of joint contractures and tissue laxity in the same patient, as well as the paradox that both gain- and loss-of-function Piezo mutations cause relatively similar phenotypes, indicates that the relationship between joint proprioception and tissue mobility is complex and may involve feedback loops between muscle function and connective tissue remodeling during development and aging.

It would be important to study patients with these and other mutations from the point of view of connective tissue biomechanics—stiffness, shear plane mobility—at various developmental stages to examine the relationship between proprioception and myofascial function.

## 8. Implications for Personalized Treatment

Despite the prevalence of myofascial pain, there is much to be learned about its clinical management. Although nonpharmacological therapies, including manual and movement-based therapies, are currently recommended as the first-line treatment for chronic, non-specific musculoskeletal pain [58], there are significant gaps in the knowledge base about how these therapies should be customized to individuals, according to whether their connective tissue are hypo- or hypermobile, and how well patients can sense their body movements. Physical therapy is underused for scleroderma, for example, because physical therapists are unsure about its safety [59]. A stretching exercise or yoga pose may be appropriate for a hypo-mobile person whose connective tissue is relatively stiff, but the same exercise could be unsafe for another person whose connective tissue is already very loose [60].

Furthermore, a person with generalized connective tissue hyperlaxity may have regional hypolaxity in some regions following injury or due to posture habits. The role of developing an improved sense of proprioceptive interoception in guiding movement is also an area in need of more research. Developing “precision” interventions based on a better understanding of fascia biomechanics and proprioception would be important to developing safer and more effective approaches to myofascial pain.

## 9. Conclusions

Fascia mobility, proprioception, and myofascial pain are three topics that, to date, have not merged in the scientific literature but have much to contribute to one another. Fascia mobility, especially along shear planes, is critical for musculoskeletal function and needs to be better integrated into biomechanical models of musculoskeletal pain. Patients with both connective tissue mobility disorders and inherited proprioception deficits suffer from musculoskeletal pain in large numbers and have poorly understood fascia biomechanics. Myofascial pain is a nascent discipline that needs to develop robust tissue biomarkers to facilitate the development of effective treatments. A better understanding of these three scientific areas will potentially synergize and result in improved care for a wide variety of patients with musculoskeletal pain.

## Data Availability

Not applicable.
